# Self-Medication Practices and Risk Factors for Self-Medication among Medical Students in Belgrade, Serbia

**DOI:** 10.1371/journal.pone.0114644

**Published:** 2014-12-11

**Authors:** Jasminka Adzic Lukovic, Vladimir Miletic, Tatjana Pekmezovic, Goran Trajkovic, Nevena Ratkovic, Danijela Aleksic, Anita Grgurevic

**Affiliations:** 1 Institute of Epidemiology, School of Medicine, University of Belgrade, Belgrade, Serbia; 2 Association for Mental Health Promotion, Belgrade, Serbia; 3 Institute of Medical Statistics and Informatics, School of Medicine, University of Belgrade, Belgrade, Serbia; 4 Clinical Centre of Serbia, Belgrade, Serbia; Baylor College of Medicine, United States of America

## Abstract

**Introduction:**

Self-medication among future health care professionals can represent a serious threat to professionalism in medicine and it has potential to put at risk public trust into this profession. The aim of this research was to investigate prevalence and risk factors for self-medication among population of medical students, because it was previously shown that their attitudes towards pharmacotherapy could affect the way they could prescribe medication in the future.

**Material and Methods:**

Research was performed as a cross-sectional study and it included 1296 (84.1%) 1st, 3rd and 6th year students of School of Medicine, University of Belgrade. Students filled out a demographic and self-medication questionnaire created for the purpose of this research and the Physical Health Questionnaire – 9 (PHQ-9). Questions about self-medication were related to the period of the previous year.

**Results:**

Self-medication was reported by 79.9% students. The most frequently self-prescribed medications were analgesics (55.4%). Independent risk factors for self-medication were possession of home-pharmacies (OR = 5.3, CI 95% 3.89–7.23), lower level of father's education (OR = 1.6, CI 95% 1.18–2.25), consumption of alcoholic beverages (OR = 1.5, CI 95% 1.13–2.08), less than 1 hour spent in physical activity per week (OR = 1.4, CI 95% 1.00–2.02), female gender (OR = 1.4, CI 95% 1.02–1.89), older age (OR = 1.1, CI 95% 1.07–1.21) and higher PHQ-9 score (OR = 1.09, CI 95% 1.05–1.12).

**Conclusions:**

Self-medication is an important issue among population of medical students. Prevalence of self-medication could be controlled through regulatory authorities and further education.

## Introduction

According to the WHO's definition, self-medication is the use of drugs to treat self-diagnosed disorders or symptoms, or the intermittent or continued use of a prescribed drug for chronic or recurrent diseases or symptoms [1].

Factors influencing frequency of self-medication in the previous studies are age, educational level, family attitudes, advertising of drug manufacturers, legislation regulating dispensing and sale of drugs, previous experiences with the symptoms or disease, significance attributed to the disease [2], [3] home-kept prescription drugs [4] and economic situation of respondents [1], [2]. Depression and anxiety may also be connected with self-medication [5].

Self-medication among university students has been surveyed in different parts of the world. In the survey conducted among final year medical students in Slovenia, 94.1% students stated that they self-medicated [6]. In the researches among students of several different universities outside Europe, prevalence ranged from 38.5% in Ethiopia [7] to 98.0% in Palestine [8]. A meta-analysis by Montgomery et al. which included 27 published studies from different countries showed that prevalence of self-medication among medical students and healthcare professionals was ranging from 12 to 99% [9]. However, no larger study has been published which explores connection between depression, living conditions and demographic characteristics to self-medication.

There are also no studies exploring self-medication among students, or general population in Serbia. The only existing study was conducted among population older than 65 years, in which 75% participants reported self-medication in the last month [10].

Survey of self-medication among student population is important because this population represents a segment of highly-educated members of the society that have better access to healthcare-related information. Of particular significance is research of self-medication among population of medical students, because they are the future generation that will have the right to prescribe drugs and to work on healthcare education. Additionally, their attitudes towards pharmacotherapy could affect the way they will prescribe medication in the future, as it was previously shown for prescribing anti-inflammatory drugs [11].

Previous research showed that medical students and healthcare professionals are facing difficulties when seeking health care for themselves [12], [13] partially because of the competitive environment they are exposed to, where commitment and regularity in studying or work, and thus good health, are required [14], [15].

The purpose of this paper is to investigate prevalence and risk factors for self-medication among population of medical university students.

## Materials and Methods

The cross-sectional study was conducted among students of the first, third and sixth year of Faculty of Medicine in Belgrade in a form of a survey that was carried out in the period December 1^st^ 2012 - March 1^st^ 2013. All interviewees voluntarily participated in the survey after being briefed in detail about the goals and methods of the study. The survey was anonymous and all obtained data is kept as confidential. The Ethical Committee of the School of Medicine, University of Belgrade reviewed and approved the study.

The survey was conducted immediately before regular classes and it consisted out of three parts.

The first part of the questionnaire included basic data about respondents (sex, age, place of residence, GPA, parental education level), personal habits (tobacco smoking, alcohol consumption, use of psychoactive substances, physical activity), health insurance, as well as data about comorbid somatic and mental disorders.

The second part of the questionnaire included data about self-prescribed medications, reasons for self-medication, methods of supply and duration of use of self-prescribed medications. All items in the questionnaire relate to the period of the previous year. The questionnaire was modelled after those used in previous studies [2], [3], [6], and it was tested on the population of thirty students, and all ambivalent and unclear questions were rephrased or removed.

The third part was the *Physical Health Questionnaire (PHQ–9)* – standardized questionnaire that estimates existence and intensity of depressive symptoms. This questionnaire includes nine items marked from zero to three by the respondents and the tenth item, which measures how the participants have handled their symptoms. Final score is obtained by summing up. Values from zero to four indicate absence of depression; from five to nine a mild depression; from 10 to 14 a moderate depression; from 15 to 19 a moderately severe depression and over 20 a severe depression [16]. This questionnaire was chosen due to it's good psychometric properties, quick administration and availability in Serbian language.

Data was analyzed using SPSS 21.0 software package. Statistical analysis was performed using descriptive statistics, χ2 test, Spearman test, Student T-test and Mann-Whitney U test to test group differences. Variables associated with self-medication at a level of significance p<0.05 were entered into the final model of the multivariate logistic regression analysis, which was used to compute adjusted odds ratio (OR) and 95% confidence intervals (95% CI) to assess the independent associations of these variables with self-medication.

## Results

The questionnaires were returned and correctly filled in by 1296 (84.1%) respondents out of total 1541 students, as follows: 472 (85.8%) from 550 first-year students, 400 (83.8%) from 480 third-year students and 424 (83.0%) from total 511 sixth-year students.

The basic respondents' demographic characteristics and the data about the respondents' living habits are presented in Table 1 and Table 2, respectively.

**Table 1:** Basic demographic characteristics and living habits of respondents.

Sociodemographic characteristics	Self-medication (Yes)	Self-medication (No)	p*
**Gender, n (%)**			**<0.001** a
males	371 (74.3)	128 (25.2)	
females	662 (83.4)	132 (16.6)	
no answer	2 (100)	0 (0)	
**Age (years), mean ± SD**	22.19±6.58	21.16±2.98	**<0.001** b
**Study year**			**<0.001** a
1^st^ year	359 (76.1)	113 (23.9)	
3^rd^ year	297 (74.4)	102 (25.6)	
6^th^ year	379 (89.4)	45 (10.6)	
no answer**	1 (100)	0 (0)	
**GPA, mean ± SD**	8.15±0.84	8.38±0.91	0.266^b^
**Level of mother's education, n (%)**			0.303^a^
primary	19 (70.4)	8 (29.6)	
secondary	408 (80.6)	98 (19.4)	
below degree	150 (86.7)	23 (13.3)	
university degree	431 (77.1)	128 (22.9)	
I don't know**	28 (90.3)	3 (9.7)	
**Level of father's education, n (%)**			**0.021** a
primary	28 (82.4)	6 (17.6)	
secondary	378 (83.1)	77 (16.9)	
below degree	112 (75.7)	36 (24.3)	
university degree	466 (77.2)	138 (22.8)	
I don't know**	50 (94.3)	3 (5.7)	
no answer**	2 (100.0)	0 (0.0)	
**Having a partner**			0.153^a^
in a relationship	516 (81.5)	117 (18.5)	
single	519 (78.4)	143 (21.6)	
no answer**	1 (100.0)	0 (0.0)	
**Housing, n (%)**			0.402^a^
in own apartment	464 (78.5)	127 (21.5)	
in leased apartment	320 (81.0)	75 (19.0)	
in a student dormitory	246 (82.0)	54 (18.0)	
other**	4 (50.0)	4 (50.0)	
no answer**	2 (100.0)	0 (0.0)	
**With whom they live, n (%)**			0.501^a^
alone	212 (81.9)	47 (18.1)	
with parents	337 (79.5)	87 (20.5)	
with roommate(s)	369 (80.4)	90 (19.6)	
with relatives	88 (75.2)	29 (24.8)	
with landlord(s) **	4 (50.0)	4 (50.0)	
with partner	22 (88.0)	3 (12.0)	
no answer**	4 (100.0)	0 (0.0)	

*statistical significance was considered at p<0.05.

** not included in the analysis.

a based on the results of χ^2^ test.

b based on the results of the Student t-test.

**Table 2:** Living habits of the respondents.

Sociodemographic characteristics	Self-medication (Yes)	Self-medication (No)	p*
**Time spent studying (daily), n (%)**			0.095^a^
1 h and less	161 (81.7)	36 (18.3)	
1–3 h	419 (81.0)	98 (19.0)	
3–5 h	324 (80.6)	79 (19.4)	
more than 5 h	129 (72.9)	48 (27.1)	
no answer**	2 (100.0)	0 (0.0)	
**Time spent in sport and physical activities (weekly), n (%)**			**0.004** a
1 hour and less	354 (85.1)	62 (14.9)	
1–5 hours	680 (77.5)	197 (22.5)	
5–10 hours	1 (50.0)	1 (50.0)	
no answer**	1 (100.0)	0 (0.0)	
**Frequency of meeting friends, n (%)**			0.615^a^
every day	188 (82.5)	40 (17.5)	
several times a week	441 (80.2)	109 (19.8)	
once a week	224 (80.0)	56 (20.0)	
once to several times a month	151 (77.4)	44 (22.6)	
less than once a month	31 (73.8)	11 (26.2)	
no answer**	1 (100.0)	0 (0.0)	
**Alcohol consumption, n (%)**			**0.019** a
yes	604 (82.2)	131 (17.8)	
no	430 (76.9)	129 (23.1)	
no answer**	2 (100)	0 (0.0)	
**Smoking, n (%)**			0.374^a^
yes	221 (81.9)	49 (18.1)	
no	814 (79.4)	211 (20.6)	
no answer**	1 (100.0)	0 (0.0)	
**Consumption of psychoactive substances (PAS)**			0.503^a^
yes	50 (83.3)	10 (16.7)	
no	963 (79.8)	244 (20.2)	
I don't know**	22 (81.5)	5 (18.5)	
no answer**	0 (0.0)	1 (100	
**Possession of home pharmacy**			**<0.001** a
yes	828 (87.6)	117 (12.4)	
no	193 (58.0)	140 (42)	
no answer**	14 (82.4)	3 (17.6)	

*statistical significance was considered at p<0.05.

** not included in the analysis.

a based on the results of χ^2^ test.

Thirty five (2.7%) respondents reported having a chronic somatic illness (mitral valve prolapse, polycystic ovaries, scoliosis and diabetes).

Eighteen (1.4%) respondents stated that they suffer from a mental disorder, 5 (0.5%) respondents specified that they suffer from depression, 1 (0.1%) stated that he/she suffers from schizophrenia and 1 (0.1%) stated that he/she suffers from bulimia, whereas 11 subjects did not specify their diagnosis.

During the previous year 1035 (79.9%) students used medications without their doctor's advice, as follows: 76.2% students of the first, 74.9% students of the third and 89.4% students of the sixth study year. The sixth-year students self-medicated more often than the first-year (χ^2^ = 26.9, p<0.001) and the third-year (χ^2^ = 29.6, p<0.001) students, whereas as regards frequency of self-medication there was no statistically significant difference between the first-year and the third-year students (χ^2^ = 0.2, p = 0.660). Out of 1035 students that self-medicated in the past year, 597 (46.1% of total number of students) self-medicated with antibiotics, antimicotics, sedatives and antidepressives, although dispensing and purchase of these drugs should be possible only with doctor's prescription. Third year students self-medicated with prescribed medication significantly less than the students of the first (χ^2^ = 4.55 p = 0.033) and sixth year (χ^2^ = 10.85, p<0.001).

Female respondents self-medicated more often than male respondents (χ^2^ = 15.54, p<0.001). Additionally, a negative correlation between self-medication and respondents' age (r = −0.13, p<0.001) was established using Spearman's test. Frequency of self-medication did not depend on mother's educational background (χ^2^ = 9.30, p = 0.363), but it depended on father's educational background, thus the respondents whose fathers had primary and secondary school educational background self-prescribed more frequently than the students whose fathers had below-degree and university degree qualification (χ^2^ = 6.02, p = 0.014). Also, there was no statistically significant difference in self-medication depending on the relationship status (χ^2^ = 2.04, p = 0.153), the place where respondents reside (χ^2^ = 1.82, p = 0.402), with whom they are living (χ^2^ = 3.35, p = 0.501), how often they are seeing their friends (χ^2^ = 2.67, p = 0.615) or how much time they spend in studying (χ^2^ = 6.38, p = 0.095). Frequency of self-medication did not depend on grade point average (GPA) (t = −2.90, p = 0.674).

Regarding physical activities, the respondents who were physically active in average less than one hour per week, self-prescribed more frequently compared to their fellow students (χ^2^ = 11.33, p = 0.003). Students who consume alcoholic beverages self-medicated more frequently (χ^2^ = 5.46, p = 0.019). This was not demonstrated for students consuming tobacco (χ^2^ = 0.79, p = 0.374) or psychoactive substances (χ^2^ = 0.45, p = 0.503).

Total of 73.9% respondents reported having a home-pharmacy. Out of them, 27.4% had home-pharmacies with five and less medicines, 30.5% between five and ten medicines, and 42.1% with more than ten medicines. The respondents who had home-pharmacies self-medicated more frequently (χ^2^ = 134.8; p<0.001), but their size did not affect self-medicating (χ^2^ = 2.54; p = 0.281).

The most frequently self-prescribed medications were analgesics (55.4%), vitamin supplements (45.7%) and antipyretics (41.5%), (Table 3). Among the most frequently self-prescribed medications, antibiotics were in the fourth place (38.9%). The first-year students (χ^2^ = 7.3, p = 0.007) and the sixth-year students (χ^2^ = 16.0, p<0.001) self-prescribed antibiotics more often than the third-year students, whereas no statistically significant difference was observed between self-medication frequency among the first-year and the sixth-year students (χ^2^ = 2.08, p = 0.149). Table 3 and 4 present an overview of self-medication frequency of certain medication groups by years and overview of self-medication frequency of certain medication groups compared to previous studies, respectively.

**Table 3:** Differences between study years and type of self-prescribed medication.

DRUG^a^	1^st^ year	3^rd^ year	6^th^ year	Total	?^2^	p
analgesics	43.4%	51.9%	72.0%	**55.4%**	76.760	.000
vitamin supplements	43.9%	45.9%	47.5%	**45.7%**	1.224	.542
antipyretics	26.9%	35.6%	63.3%	**41.5%**	130.285	.000
antibiotics^b^	40.0%	31.3%	44.7%	**38.9%**	15.912	.000
decongestants	25.2%	21.1%	33.9%	**26.8%**	18.202	.000
mineral supplements	15.3%	16.8%	19.3%	**17.1%**	2.608	.271
histamine antagonists	12.7%	12.3%	19.1%	**14.7%**	9.810	.007
herbal preparations	12.3%	13.0%	16.7%	**14.0%**	4.050	.132
sedatives^b^	7.2%	12.8%	19.8%	**13.0%**	31.152	.000
antidiarrhoeals	10.2%	9.3%	17.6%	**12.3%**	16.584	.000
corticosteroids	8.9%	7.3%	13.9%	**10.0%**	11.031	.004
antimycotics^b^	5.9%	7.0%	9.4%	**7.4%**	4.075	.130
“morning after” pills	7.2%	6.8%	5.4%	**6.5%**	1.262	.532
oral contraceptives	5.5%	3.5%	5.2%	**4.8%**	2.112	.348
laxatives	5.3%	4.8%	1.4%	**3.9%**	10.369	.006
supplements for body mass reduction	3.4%	2.0%	0.7%	**2.1%**	7.915	.019
antidepressants^b^	4.2%	1.0%	0.5%	**2.0%**	19.094	.000
anabolic steroids	1.1%	1.8%	0.2%	**1.0%**	4.806	.090

a 1.9% respondents additionally stated that they did not know what medications they had self-prescribed.

b prescription-only drugs.

**Table 4:** Self-medication by drug groups and comparation with other studies on students.

DRUG^a^	Our study (%)	Previous studies (%)	Reference
analgesics	**55.4**	81.3–87.2	[2] b, [8], [25], [33]
vitamin supplements	**45.7**	11.1–54.4	[24], [33], [34]
antipyretics	**41.5**	74.8	[33]
antibiotics^c^	**38.9**	6.0–58.8	[2] b, [8], [25], [33], [34]
decongestants	**26.8**	12.7–45.3	[2] b, [8],
mineral supplements	**17.1**	N/A	
histamine antagonists	**14.7**	6.6–41.6	[8]
herbal preparations	**14.0**	17.0–32.4	[8], [25], [35]
sedatives^c^	**13.0**	12.0–29.0	[25]
antidiarrhoeals	**12.3**	18.7–30.6	[8]
corticosteroids	**10.0**	18.0	[8]
antimycotics^c^	**7.4**	N/A	
“morning after” pills	**6.5**	N/A	
oral contraceptives	**4.8**	N/A	
laxatives	**3.9**	<1	[36]
supplements for body mass reduction	**2.1**	7.8	[37]
antidepressants^c^	**2.0**	N/A	
anabolic steroids	**1.0**	3.3	[38]

a 1.9% respondents additionally stated that they did not know what medications they had self-prescribed.

b only first year students.

c prescription-only drugs.

Most frequently, respondents decided what medication they would use for self-treatment based on their own knowledge and experience (56.2%) and this was mostly done because the symptoms of their disease were not serious (60.1%). Most frequently they purchased medications in pharmacies (74.6%).

In most cases students used self-prescribed medications for one week or shorter (92.2%), with no statistically significant difference among study years (χ^2^ = 3.0, p = 0.221). In total, 1% of respondents reported an adverse reaction after the use of self-prescribed medication, out of which 0.6% stated allergic reaction, while others did not specify what adverse reaction they've had. That taking medications without doctor's advice is never dangerous think 2.5% students, while 86.0% students think that it depends on the medicine and 11.4% respondents think that it is always dangerous to take medications without doctor's advice.

During the previous year, 6.5% of respondents altered dosage of medicine prescribed by their doctors. By analyzing the duration of taking the prescribed drugs it was observed that 27.9% respondents took their medicine for a shorter period of time, and 5.5% respondents took their medicines for a longer time periods than prescribed. The first-year students corrected the dosage more frequently than the third-year students (χ^2^ = 8.0, p = 0.005) and the sixth year students (χ^2^ = 4.4, p = 0.035) and took their therapy more often for shorter periods of time than prescribed compared to the third-year students (χ^2^ = 13.3, p<0.001) and the sixth-year students (χ^2^ = 4.0, p = 0.047). As regards frequency of taking the prescribed therapy for a longer time than prescribed no difference between study years was observed (χ^2^ = 2.1, p = 0.341).

Average value of PHQ-9 questionnaire score was 6.21±4.96, and the values ranged from 0 to 27. Potentially clinically significant symptoms of depression were reported by approximately half students (55.1%), and the highest number (33.0%) among them had a mild depression, 14.9% moderate, 5.0% moderately severe and 2.2% severe depression. Male respondents had slightly higher average score (6.41±5.29) than female students (6.10±4.73), but this difference is not statistically significant (U = 195304.00, p = 0.679), as demonstrated by Mann-Whitney U test. It was shown by the same test that respondents who were physically active in average less than one hour per week, had higher PHQ-9 score compared to their fellow students.

Data analysis showed that the students who self-medicated during the previous year had statistically significant higher PHQ-9 score than the students who did not self-medicate (U = 106111.50, p<0.001), what was established for the students of the first (U = 15026.50, p<0.001), third (U = 12456.50, p = 0.022) and sixth year (U = 5908.50, p = 0.001) individually. Also, during the analysis it was established that the students who tolerated their symptoms with more difficulties also self-medicated more frequently (t = 4.56, p<0.001).

Multivariate logistic regression analysis was performed in order to single out independent risk factors for self-medication among medical students, as shown in Table 5. Variables which were independently associated with self-medication were: possession of home-pharmacy (OR = 5.30, p<0.001), female gender (OR = 1.39, p = 0.035), older age (OR = 1,13, p<0.001), lower level of father's education (OR = 1.63, p = 0.003), consumption of alcoholic beverages (OR = 1.53, p = 0.007), higher PHQ-9 score (OR = 1.09, p<0.001) and lower level of physical activity (OR = 1.42, p = 0.049).

**Table 5:** Multiple logistic regression model with self-medication as the dependent variable.

Independent variables	B	Wald	p	Odds ratio	95% C.I. for odds ratio
possession of home-pharmacy (yes/no)	1.67	111.27	**<.001**	**5.30**	3.89–7.23
level of father's education (primary and secondary school/higher education)	0.49	8.77	**.003**	**1.63**	1.18–2.25
consumption of alcohol beverages (yes/no)	0.42	7.36	**.007**	**1.53**	1.13–2.08
physical activity less than 1 hour per week	0.35	3.83	**.049**	**1.42**	1.00–2.02
gender (female/male)	0.33	4.43	**.035**	**1.39**	1.02–1.89
age	0.13	16.52	**<.001**	**1.13**	1.07–1.21
PHQ-9 score	0.08	21.57	**<.001**	**1.09**	1.05–1.12

Multivariate logistic regression was also performed in order to define independent risk factors for self-medication with prescription-only drugs, as shown in Table 6. Variables which were independently associated with self-medication of prescription-only drugs were: studying 1 h or less a day (OR = 1.69, p = 0.020), tobacco consumption (OR = 1.80, p = 0.002), high level of mother's education (OR = 1.46, p = 0.023) and PHQ-9 score (OR = 1.11, p<0.01).

**Table 6:** Multiple logistic regression model with self-medication of prescription-only drugs as the dependent variable.

Independent variables	B	Wald	p	Odds ratio	95% C.I. for odds ratio
tobacco consumption (yes/no)	0.59	9.87	**.002**	**1,80**	1.25–2.59
studying for 1 h or less per day	0.53	5.39	**.020**	**1.69**	1.08–2.63
level of mother education (high education/primary, secondary, higher education)	0.38	5.18	**.023**	**1.46**	1.05–2.02
PHQ-9 score	0.10	28.46	**<.001**	**1.11**	1.07–1.15

## Discussion

In our survey, 79.9% respondents used medication without doctor's advice during the previous year and 46.1% of total number of students self-medicated with prescription-only drugs. This was done more frequently by the students in the final, sixth year, where 89.4% students self-medicated and 62.8% self-medicated with prescription-only drugs. Due to lack of studies concerning self-medication among general population in Serbia, the level of self-medication is difficult to rate.

In our survey, female respondents self-medicated about 1.4 times more frequently compared to male respondents. This might be due the fact that women seem to perceive drugs as more powerful and to believe that prevention and treatment are more effective than men [17]. Research conducted among students in Slovenia [6] and Palestine [18] did not show statistically significant differences among genders, while some surveys, nevertheless showed that male students [19] or female students [2] statistically significantly self-medicate more frequently.

Older students self-medicated more than younger students, as it was shown in other studies [2], [3], [20]. For each year of age, there was about 1.13 times more chance to self-medicate. The fact that 6^th^ year students chose drugs for self-medication based on their own knowledge and experience, might be due the fact that older students believe that they have enough knowledge to self-diagnose and self-medicate without additional exams.

Students whose fathers had only primary and high school education had 1.6 times greater chance to self-medicate than students whose parents had higher education. Previous studies proved that family attitudes play a role in the prevalence of medication use and these are in many ways determined with societal values and norms [2], in case of Serbia still very conservative and patriarchal. On the other hand, a high level of education and professional status of mothers are showed to be risk factors for adolescent self-medication in some studies [21]. In our study, this finding was only true for self-medication with prescription-only drugs, where students whose mother's had a high level of education self-medicated about 1.5 times more often.

Since previous researches have indicated that self-medication with drugs can predicate higher risk of new-onset drug and alcohol abuse among those with baseline mood disorders [22] and that people that self-medicate can get addicted due to self-medication [23], we included questions about consumption of alcohol, tobacco and psychoactive substances. Respondents in our study who consumed alcoholic beverages self-medicated about 1.5 times more frequently than non-drinking respondents and tobacco consumers self-medicated about 1.8 more than non-tobacco consumers, but this did not apply to use of psychoactive substances. In interpretation of these results, it is important to mention that alcohol consumption and tobacco use are considered as socially acceptable behaviour in Serbia, while there is stigma concerning consumption of psychoactive substances [24]. Due to the fact that this might have lead to social desirability bias and that our surveys were rather vague on the criteria for substance abuse, alcohol and tobacco consumption, interpretation of these result results requires further research.

Most frequently self-prescribed medications are analgesics (55.4%), vitamin supplements (45.7%) and antipyretics (41.5%). Analgesics and antipyretics are among the most frequently self-prescribed medications in other surveys as well [7], [8], [18], [25], [26] and in significant number of surveys antibiotics are among the first five most frequently self-prescribed medications with self-medication rate over 30% [8], [18], [25]–[27].

In our study antibiotics were self-prescribed by 38.9% respondents and there were most frequently self-prescribed drugs which dispensing and purchase are possible only with doctor's prescription. Compared to their fellow students, these medicines were self-prescribed in a lesser amount by the third-year students. Total self-prescription rates of both over the counter drugs and prescription-only drugs were also significantly smaller in the population of the third year students, maybe due to the first pharmacological course that begins in the third year, when students learn about interactions and adverse effects for the first time.

Sedatives were self-prescribed by each tenth interviewed medical student (13.0%) and almost by every fifth sixth year student (19.8%). Previous studies among students showed that self-medication with stimulants, sedatives and sleeping medications was connected with self-perceived academic load and stress [28]. The fact that respondents in our study had most frequently bought their medication in pharmacies, as had the students in most previous studies [7], [18], [25], [29] suggests that legal obligations might not be obeyed and issuing of these drugs controlled.

As in our study, surveys conducted in other countries also show that most frequently students choose what medicine they would self-medicate based on their own knowledge and experience [4], [6], [7], [28], [30].

In our research, respondents mainly chose to self-medicate because the symptoms of their disease were not serious. This was also confirmed in other studies [7], [22], [25], [30]. However, long waits at the doctor's office were the reason why almost one fifth of our respondents (19.8%) chose to self-medicate.

Students who spent less than one hour in physical activity per week, self-medicated about 1.4 times more often than their fellow students. To our knowledge, this factor was not investigated in other studies concerning self-medication. This result, in a country in which 70.9% inhabitants are inactive [31], among other data, raises a question of introducing some programs that would aim on increasing the level of weekly physical activity, especially because other studies have previously proven that physically active students have better quality of life [32].

Respondents who on average studied less than 1 hour a day self-medicated with prescription-only drugs approximately 1.7 times more. Due to the nature of medical studies, this could be explained with the fact that students who study more could be more responsible than the students that don't study regularly.

The respondents who had reported having home-pharmacies self-medicated about 5.3 times more often than their colleagues. Home-pharmacies were shown to be correlated with self-medication in other studies, as well [4], [19].

About one half of the students (55.1%) in our research showed symptoms of depression. Also, the students who self-medicated during the previous year had significantly higher depression symptoms scores than the students who did not self-medicate. For every PHQ-9 point, risk was about 1.1 point higher and this also applied for self-medication with prescription-only drugs. Depression was also correlated with self-medication in another study, but among students of veterinary medicine, where depressive symptoms were measured by Hamilton scale [5].

Our study has several limitations. First, since this was a cross-sectional study, each variable was measured only once and exposure and outcome are simultaneously assessed, so evidence of any associations should be closely interpreted before a causal relationship could be established. Second is the absence of comparative groups, like students from other faculties. Third, the study is based on self-reported data and it comprises questions for the period of the previous year, thus it is possible that some incorrect data was given due to forgetfulness, or so called recall bias. Information bias should be also taken into account, because of stigma concerning consumption of psychoactive substances [24]. Fourth, although the students were encouraged to fill in the questionnaire independently, mutual influences among respondents cannot be fully excluded. However, this study included a representative sample of the population of medical students, due to high response rates.

Self-medication is an important issue among population of medical students. Self-medication in the research sample is connected with female gender, older age, lower level of father's education, consumption of alcoholic beverages, possession of home-pharmacies, physical inactivity and depressive symptoms and their tolerability. In addition to self-medication, almost every third respondent displayed another form of risky behaviour when using medications – taking medication for a shorter period than prescribed by his/her doctor. From the results, it can also be concluded that strict legal regulations concerning control of selling potentially dangerous medications should be implemented. Further education regarding this issue should also be taken into consideration, because self-medication among future healthcare professionals can represent a serious threat to professionalism in medicine and it has potential to put at risk public trust into this profession.
